# COVID-19 patient and personal safety – lessons learnt for pandemic preparedness and the way to the next normal

**DOI:** 10.1186/s13756-023-01231-1

**Published:** 2023-04-01

**Authors:** Astrid Füszl, Julia Ebner, Miriam Van den Nest, Lukas Bouvier-Azula, Magda Diab-El Schahawi, Elisabeth Presterl

**Affiliations:** Department of Infection Control and Hospital Epidemiology, Medical University of Vienna, Vienna, Austria

**Keywords:** COVID-19, Infection prevention and control, Healthcare-associated transmission, Hospital cluster, Pandemic preparedness, Pandemic response

## Abstract

**Background:**

The COVID-19 pandemic has profoundly challenged societies and healthcare systems in particular. To prevent the spread of SARS-CoV-2, infection prevention and control (IPC) strategies had to be developed on the local, national and international level. The aim of this study is to provide details of the COVID-19 experience at the Vienna General Hospital (VGH) in the context of the national and international COVID-19 response for learning and improvement.

**Methods:**

This is a retrospective report, outlining the evolution of IPC measures and challenges encountered at the health facility (VGH), the national (Austria) and global level between February 2020 and October 2022.

**Results:**

The IPC strategy at the VGH has been continuously adapted to changes in the epidemiological setting, new legal directives and Austrian by-laws. The current strategy, nationally and internationally, focuses on endemicity rather than maximum transmission risk reduction. For the VGH, this has recently resulted in an increase in COVID-19 clusters. To protect our particularly vulnerable patients, many COVID-19 precautions have been maintained. Barriers to adequate IPC implementation at the VGH and other hospitals include a lack of sufficient isolation options and non-adherence with universal face mask regulations. Globally, misinformation on COVID-19 hampered an effective response.

**Conclusions:**

This retrospective analysis of the COVID-19 response at the VGH and international reports underline the need for pandemic preparedness, readiness and response by improving future hospital design and infrastructure, conducting regular trainings for protective attire and increasing health literacy as now recently published in a concise document by WHO.

**Supplementary Information:**

The online version contains supplementary material available at 10.1186/s13756-023-01231-1.

## Background

In December 2019, a new coronavirus later termed severe acute respiratory syndrome coronavirus 2 (SARS-CoV-2) emerged in Wuhan, China and has since spread across the globe, causing the novel coronavirus disease 2019 (COVID-19) pandemic. Worldwide, countries and hospitals have been trying to contain the spread of the virus by drafting infection prevention and control (IPC) regulations. In the absence of adequate COVID-19 precautions, in-hospital infection with SARS-CoV-2 poses a major problem for healthcare facilities [1, 2], causing excess morbidity and mortality especially among patients with certain underlying medical conditions [3, 4]. Hospitals have been pursuing different IPC strategies, depending on the available resources and the given hospital infrastructure. Testing strategies have been ranging from universal pre-admission screening [5] and periodic re-testing [6–8] to targeted screening (e.g. prior to aerosol-generating procedures), or testing of symptomatic individuals only [9]. Isolating a large number of patients was not feasible in many institutions due to the lack of single rooms [10]. Routine testing of healthcare workers (HCWs) has not been universally applied. For example, Singapore implemented rostered twice-weekly testing of HCWs in acute care hospitals as a response to a surge in cases caused by the emergence of more transmissible variants [11]. Similarly, universal face mask/N95 respirator use – though recommended by public health authorities [12] – was not implemented by all institutions or was employed at later pandemic stages as a means of controlling hospital clusters [13].

In response to the pandemic, the WHO developed and published an elaborate IPC document for readiness, preparedness and response to outbreaks and epidemics [14].

In this report, we describe the preventive measures at Vienna General Hospital (VGH) as well as the Austrian and international level in response to different stages of the pandemic.

## Methods

This is a descriptive retrospective study summarizing the different phases of the COVID-19 pandemic and the corresponding IPC measures at the hospital, national and global level between February 2020 and October 2022. It is divided into four chapters: [1] “Early phase” (February-April 2020), [2] “Several COVID-19 waves” (August 2020-January 2022), [3] “The spread of Omicron” (February 2022-July 2022) and [4] “Present situation” (October 2022). For the VGH, additional information on COVID-19 clusters and context-specific challenges encountered during IPC implementation is provided.

### Definitions

A case of confirmed SARS-CoV-2 infection is defined as a person with a positive nucleic acid amplification test (NAAT), regardless of clinical or epidemiological criteria [15].

A fully vaccinated individual is defined as somebody who has received a total of three vaccinations (immunocompetent individual), administered in the scheme 2 + 1 (2 initial vaccinations plus a further vaccination after 6 months). After the basic immunization, further booster vaccinations are necessary. Past infection with SARS-CoV-2 does not count as a replacement of these vaccinations [16].

A cluster/an outbreak is defined as at least two cases of confirmed COVID-19, with one patient becoming positive seven or more days after admission and an epidemiological link between these cases (overlap on the same unit or ward) [17]. In this report, only clusters affecting more than three patients will be presented.

### Setting

Austria is a federal state consisting of nine provinces with a population of 8.9 million. The Austrian Ministry of Health (MOH) released and regularly updated a number of guidance documents (e.g. handling of COVID-19 cases and their contacts). Both the state and the provinces have certain legislative mandates, which resulted in partially divergent provincial preventive approaches.

The VGH is the biggest academic tertiary hospital in Austria with a total capacity of 1,738 beds (134 ICU beds) and 8,979 employees. It is a competence center for oncology, heart and lung transplantation, advanced neonatal intensive care and a referral center for rare diseases. The single room capacity is 3%. The remaining rooms are two- or three-bed-rooms. An overview map of the VGH is presented in Additional file 1: Figure S1 (Supplement).

## Results

### Early phase of the pandemic (February-April 2020)

#### The VGH: temporary suspension of elective procedures

Initially, non-urgent treatment of in- and outpatients was suspended while emergency care was provided as usual. In April/May 2020 - alongside a very low COVID-19 incidence due to draconic national public health regulations - the VGH decided on a gradual return to full medical operations to reduce collateral damage and excess mortality from COVID-19-unrelated causes [3, 4]. The IPC concept was based on international and local guidance documents [18, 19]. The main interventions to contain the in-hospital spread of SARS-CoV-2 are summarized below. To keep it simple these interventions were nearly the same at normal ward and ICUs but for specific procedures there were written workflows. The “Additional file 1: Table S1 (Supplement)” contains more detailed information on all measures taken.

A general visitor ban was introduced with only few exceptions (e.g. terminal care patients, children). These visitors had to be clear of any COVID-19 related symptoms and without recent exposure to a COVID-19 case. The use of a medical face mask was mandatory inside the hospital.

The hospital’s management and IPC teams set up an elaborate testing strategy for elective patients, acute patients, staff and visitors [20], enabled by the widespread availability of PCR tests [21]. Briefly, universal pre-admission screening was introduced, and separate testing sites established for symptomatic and asymptomatic patients. Patients requiring acute care were preventively isolated until a negative SARS-CoV-2 PCR result was available. In case of acute interventions (e.g. emergency surgery), COVID-19 precautions (see section on personal protective equipment below) were applied. Healthcare workers (HCWs) and gradually all staff involved in patient care started to be tested on a weekly basis in April/May 2020 (nasopharyngeal sample, PCR).

To allow for the legally required distance of at least two meters, 3-bed-rooms were used as 2-bed rooms. Patients undergoing aerosol-generating procedures (AGPs) were – whenever possible – placed in a single room. During hospitalization, patients were monitored for the onset of COVID-19 related symptoms including twice-daily temperature checks. Outside their designated rooms, patients had to wear a medical face mask.

At this initial stage, there was a strict separation between COVID-19 and non-COVID-19 wards for normal and ICU wards. The duration of isolation of COVID-19 cases was 14 days according to international recommendations and national regulations.

In-hospital contacts of COVID-19 cases (e.g. shared patient room) were either discharged to self-quarantine at home or isolated or cohorted at the hospital for 14 days following the exposure.

During COVID-19 patient care, HCWs used single use gloves, a single use long-sleeved gown, a face shield/goggles, a surgical cap and an appropriate mask. Mask requirements changed over time (initially N99 respirators during COVID-19 patient care, downgraded to N95 respirators in March 2020) [22]. For the care of patients not suspected of having COVID-19, medical face masks were initially used. For staff training, a video on the correct donning and doffing of personal protective equipment (PPE) was released, accompanied by numerous face-to-face trainings delivered by the hospital’s IPC team. Hospital staff received regular email updates on new measures of containment and any changes of policy.

Environmental cleaning of rooms occupied by COVID-19 patients followed the hospital’s routine procedures. After patient discharge, these rooms were deep-cleaned (cleaning and disinfection of all surfaces, including the walls).

#### Clusters

No clusters were recorded during this period.

#### Austria: nationwide lockdown

After the first two cases of Covid-19 were confirmed in Austria on February 25th 2020, numbers quickly increased. The Austrian government responded with a nationwide lockdown middle of March. Leaving one´s home was only allowed for a handful of reasons (e.g. certain jobs, grocery shopping, assisting other people in need).

Once shops, schools and restaurants reopened again, preventive measures such as social distancing and the use of face masks (e.g. on public transport, in supermarkets and healthcare settings) were introduced. Large gatherings were prohibited.

At this early stage, only people who were both (a) symptomatic and (b) met specific criteria (e.g. admitted to a hospital, previously exposed to a known case, travelers returning from areas considered high-risk) were tested. By the end of March, anyone presenting with symptoms according to the case definition was tested. Alongside, contact tracing was established.

#### International level: slow response and lack of coordination

WHO declared the outbreak a Public Health Emergency of International Concern on January 30th, 2020. Most countries – apart from the ones in the Western Pacific region who had been better prepared through their experience with the SARS-CoV-1 outbreak – reacted reluctantly [23].

### Pandemic waves from August 2020 to January 2022

#### The VGH: nearly normal hospital operation despite high case numbers and regular adaptations of the initial IPC strategy

During this period, there was a slowly rising and then high COVID-19 incidence. The concurrent strategy was “back to business as usual” with a high patient turnover, necessitating an escalation of IPC measures.

In November 2020, the universal use of N95 respirators was introduced for all areas of VGH. Further, the testing frequency of inpatients was increased (re-testing during hospitalization in addition to pre-admission screening).

By now, due to the lack of sufficient isolation capacities, patients presenting with acute conditions were admitted to a multi-bedroom with a negative antigen test result only, preferably equipped with an N95 respirator until confirmed SARS-CoV-2 negative by PCR. To cope with increasing room demands for COVID-19 patient care, mixed wards were established in addition to designated COVID-19 wards. There, SARS-CoV-2 positive patients received treatment for conditions unrelated to COVID-19. The initial strategy regarding the isolation of COVID-19 cases was adapted in summer 2020, and an early termination of preventive measures was now possible after 10 days.

The number of allowed visitors was limited. They were required to wear an N95 respirator and had to provide a negative SARS-CoV-2 test and/or proof of full vaccination or recent recovery.

It was legally required for all newly employed HCWs to be vaccinated with an approved COVID-19 vaccine (if there were no contraindications). In late May 2021, the screening of HCWs by means of self-testing was introduced (gargle test, PCR). The required testing frequency varied, ranging from once per week during low incidence phases to daily testing during the high incidence periods. Vaccinated personnel was not exempt from testing.

The major challenges for IPC implementation and coping strategies are summarized in Table 1.

**Table 1:** Context-specific IPC challenges and coping strategies due to COVID-19 at the Vienna General Hospital

Challenges	Coping strategies
Early phase	
PPE shortages	• Conditional re-use of masks (no visible contamination, person-related, etc.) • Use of medical face masks instead of N95 respirators (both patient and HCW equipped with a medical face mask) • Strategy for re-processing masks via steam-sterilization (never came into effect)
Shortages of disinfectants	• In-house production of hand disinfectant by the hospital’s pharmacy • Purchase of products with a comparable activity spectrum for surface disinfection
**Several COVID-19 waves**	
Limited isolation capacities for suspected cases at the emergency department: only one separate room available for the assessment/management of cases presenting with respiratory symptoms	• Prioritization of PCR test analysis at the laboratory to quickly obtain a result • Universal N95 mask mandate for all patients (whenever possible)
Few isolation options in outpatient departments	• Blocking of seats in waiting areas to ensure enough space between waiting patients • Universal N95 mask mandate
Limited single room capacities	• Extensive testing strategy (screening of patients prior to admission, routine re-testing during hospitalization)
Doors to patient rooms could not be kept closed at all times due to agitated and/or confused patients or lack of monitoring equipment for unstable patients	• Universal N95 mask requirements for healthcare personnel • Frequent testing of patients
Lack of sufficient airborne infection isolation rooms (AIIRs) for confirmed cases	• Doors should remain closed
Labor-intensity of entire PPE change between COVID-19 patients – only plastic apron was changed	• Continued surveillance and heightened vigilance regarding outbreaks from patients colonized/infected with multidrug resistant organisms on COVID-19 wards
Adherence issues regarding universal respirator use among hospital staff (e.g. masks removed by HCWs in recreational areas)	• Regular reminders to adhere to the in-house regulations • Frequent SARS-CoV-2 screening of HCWs
Low compliance with universal N95 respirator mandate among visitors	• Visitor restrictions • Requirement to show proof of a low transmission risk upon entry (e.g. prior vaccination/infection/recent negative test result)
Introduction of combined screening tests for Influenza/SARS-CoV-2/RSV. How to proceed with incidental findings, e.g. asymptomatic RSV positive patients?	• Isolation of RSV positive cases in high-risk areas (obstetrics, neonatal, pediatrics departments)
**The spread of Omicron**	
Staff shortages due to sick leave	• Shortening of quarantine to five days with a negative SARS-CoV-2 test result (or Ct-value > 30) and no COVID-19 related symptoms
Overburdened contact tracing task force	• Daily PCR screening of HCWs, accompanied by an omission of contact tracing among HCWs • Nota bene: Patients exposed to COVID-19 cases were still traced and isolated

#### Clusters

During this period, three hospital clusters were reported (02/2021: eight patients and one HCW from an internal medicine ward; 10/2021: eight patients and five HCWs from an internal medicine ward, 11/2021: five patients and three HCWs from orthopedics/trauma surgery).

#### Austria: lockdowns, extensive SARS-CoV-2 testing, vaccination and the use of N95 respirators as essential components of the Austrian prevention strategy

After a low-incidence period during summer 2020, Austria encountered several COVID-19 waves between August 2020 and January 2022. The government responded with lockdowns, ranging from full national lockdowns to the closure of (or access restrictions for) restaurants, museums and leisure facilities only. Additional IPC measures included an extensive testing strategy, allowing all Austrians to get unlimited PCR-tests for free (screening and diagnostic testing), and mandatory masking (N95 respirators) in public areas. In January 2021, Austria launched its COVID-19 vaccination campaign. In the following months, vaccination rates were unsatisfactory due to widespread vaccine hesitancy. Hence, areas of public life were – for a period of time – restricted for unvaccinated individuals, only granting access with proof of vaccination, recovery or a recent negative PCR test. In February 2022, a general vaccine mandate came into effect, which was later repealed on grounds of disproportionality.

#### International level: different approaches and an unequal distribution of supplies and vaccines

The COVID-19 response strategies of different governments varied in terms of their scale, scope, strictness and timeline of their implementation [24, 25]. While China, for example, used a law-based top-down approach, Sweden opted for a nudge strategy without imposing mandatory restrictions on its citizens [24]. Some countries encountered substantial vaccine hesitancy and non-compliance with the use of face masks/social distancing measures [25, 26]. While the virus spurred efforts to rapidly develop vaccines, access to these vaccines was marked by inequity, disadvantaging low-income countries. This lack of widespread vaccination may have been a facilitator for the virus to evolve and persist [23].

A summary of recommendations published by international public health authorities is presented in Additional file 1: Table S2 (Supplement).

### The rise and spread of “Omicron” (February 2022-July 2022)

#### The VGH: roll-back of some measures while maintaining others

Increasing vaccination coverage and the typically more benign disease course of the Omicron variant resulted in fewer ICU admissions with severe COVID-19 [27, 28]. However, enhanced transmissibility and partial immune escape [29, 30] led to a high caseload of SARS-CoV-2 positive patients admitted for conditions unrelated to COVID-19.

Daily SARS-CoV-2 self-testing was introduced for HCWs. Sick leave due to COVID-19 led to staff shortages, resulting in the cancellation of elective procedures at the end of March 2022. Nevertheless, SARS-CoV-2 positive HCWs were allowed to resume work at earliest after five days with a negative PCR test (or a Ct-value > 30) and the resolution of symptoms. In contrast to the other Austrian provinces, Vienna maintained its policy of visitor restrictions. Patients with COVID-19 and all patients with prior exposure to a COVID-19 case (irrespective of their vaccination status) were still isolated/cohorted.

PPE requirements were downgraded, meaning that without direct contact to a COVID-19 case, hospital staff now only required an N95 respirator (instead of the full PPE) when entering the room of a COVID-19 patient.

#### Clusters

During this period, four hospital clusters were reported (03/2022: seven patients at an internal medicine ward; 03/2022: seven patients and seven HCWs at an internal medicine ward, 04/2022: nine patients at the trauma surgery ward; 06/2022: nine patients at a psychiatry ward).

#### Austria: increasing vaccination rates, a milder COVID-19 disease course and relaxation of IPC measures despite peaking case numbers

The loosening of restrictions in more and more European countries without detrimental outcomes and increasing vaccination coverage among Austrians sparked a relaxation of the national public health strategy despite a peak in cases. Most notably, the duration of quarantine for infected individuals with a mild disease course was reduced to five days. In addition, people who had received three doses of approved COVID-19 vaccines were no longer considered as contacts of COVID-19 cases, thus abrogating the mandatory home quarantine. In April 2022, MOH announced an end to Austria´s free-tests-for-all strategy, restricting the number of free tests for screening purposes to five antigen and five PCR tests per month per citizen.

**Fig. 1:**
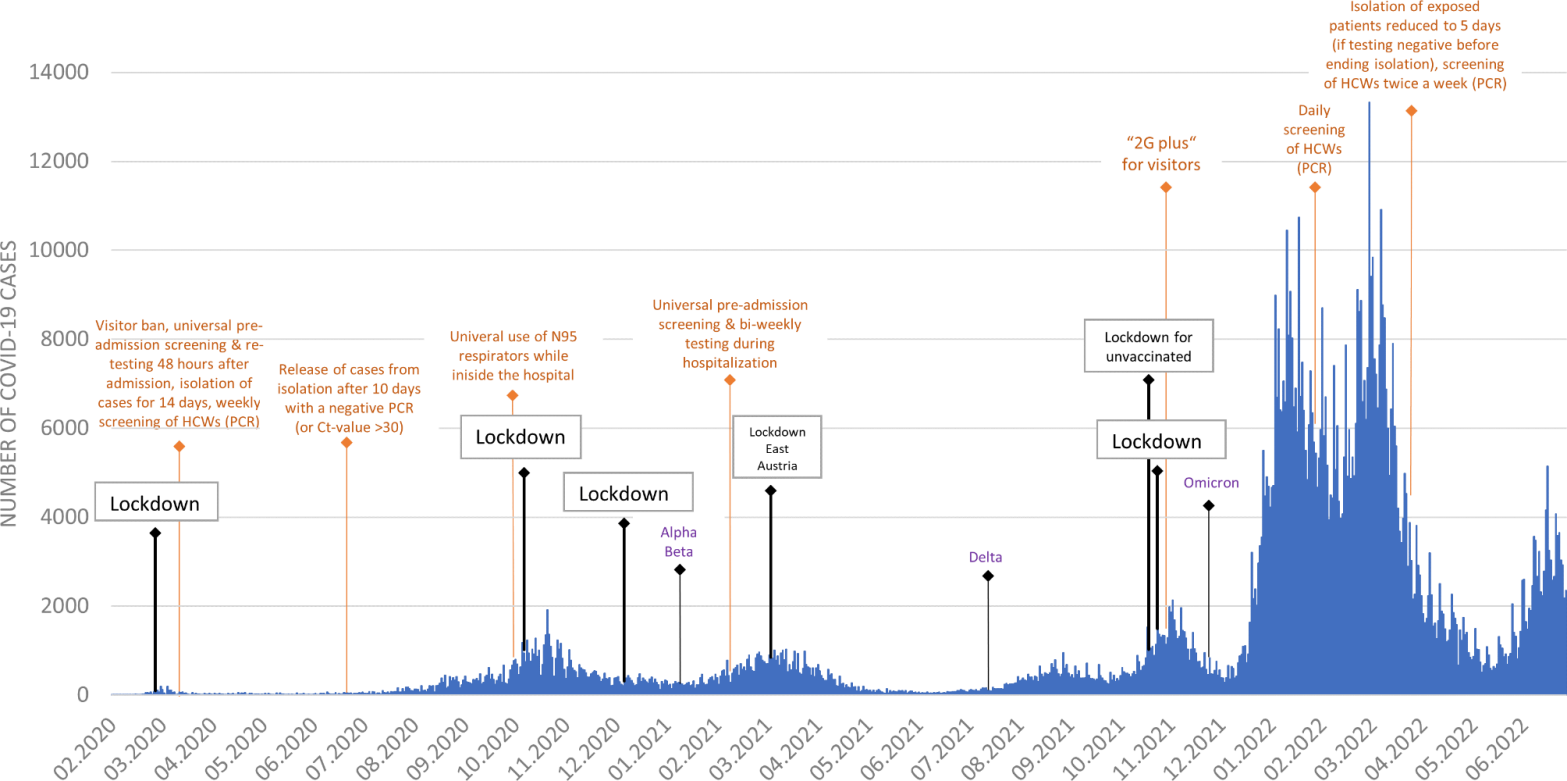
Epidemic curve (laboratory-confirmed COVID-19 cases) in Vienna and IPC measures in Austria (black colour) as well as the Vienna General Hospital (orange colour)

A summary of virus dynamics in Vienna between March 2020 and July 2022 and the preventive measures taken in response (at the hospital and state level) is presented in Fig. 1.

#### International level: back to normality

In many countries, public health measures were relieved during the Omicron waves because the COVID-19 illness was usually mild, and due to high rates of immunity from recovery and vaccination [31]. The scientific community disagreed on whether the lifting of these measures had been set prematurely [31].

### Present situation (September/October 2022)

#### The VGH: The ongoing need to protect vulnerable patients from SARS-CoV-2

The universal masking policy with N95 respirators is still in place. Patients with COVID-19 and their contacts are still isolated. The VGH does not allow healthcare staff testing positive for SARS-CoV-2 to come to work despite the end of compulsory home quarantine for the general population. Electively admitted patients and visitors still need a negative SARS-CoV-2 PCR test to be granted access. HCWs have to self-test twice a week.

#### Clusters

Recently, there have been multiple transmission clusters, either due to initially PCR-negative patients being admitted to the VGH while incubating COVID-19, SARS-CoV-2 positive visitors not wearing their masks or confused patients (e.g. with dementia) leaving their rooms without a mask (09/2022: five patients at the urology ward and four of hospital staff; 10/2022: 12 patients at the trauma surgery ward and 0 HCWs, 10/2022: at an internal medicine ward affecting 11 patients and three HCWs).

#### Austria: abandonment of most COVID-19 measures

August 1st 2022 marked the end of compulsory home quarantine of SARS-CoV-2 positive individuals in Austria. Contact tracing was suspended. Individuals with SARS-CoV-2 can now leave their homes with an N95 respirator. However, SARS-CoV-2 positive visitors are not granted access to settings with vulnerable individuals (hospitals and nursing homes).

#### International level: balancing the pandemic with other important health needs

There is still a need for sustained action against COVID-19, ensuring high vaccination coverage, the availability of tests, PPE and treatment. In case of high transmission rates, preventive public health measures like social distancing and the mandatory use of face masks may be re-installed complementarily. Health goals as defined by the Sustainable Development Goals (SDGs) are now back on the agenda [23], however still with a considerable background activity of COVID-19.

## Discussion

IPC and Public Health have gained a profound acknowledgement during this pandemic. The initial relative unpreparedness of institutions, the exploding number of COVID-19 cases, supply shortages, the lack of existing healthcare infrastructure to isolate patients, the lack of education and training in IPC practices and the relentlessness of the pandemic – all these factors contributed to an overburdening of institutions and people, particularly in healthcare [32, 33]. However, during the past 2.5 years, there has also been an incredible increase in knowledge about the SARS-CoV-2 virus, its transmission and effective prevention strategies. This report aims to summarize the local, national and international COVID-19 experience, which will enable learning from shared experiences to foresee future IPC challenges and preemtively develop mitigation strategies.

To curb the spread of this novel virus, a multimodal IPC approach has been adopted by many countries [24, 34]. The specific regulations in each country have been shaped by cultural, political, social and financial factors [24]. At the hospital level, adequate IPC strategies play a particularly important role because patients with pre-existing conditions are more likely to develop severe COVID-19, and close contact between patients and HCWs increases the transmission risk [35, 36]. There have been many reports on the use of various strategies to prevent in-hospital SARS-CoV-2 transmission among patients, visitors and HCWs [5–9, 11, 13, 36, 37]. The VGH’s strategy to contain SARS-CoV-2 transmission was continuously adapted to an ever-changing epidemiological and public health situation. There is no lasting immunity after infection [38], but the adapted vaccines are helpful for protecting against severe COVID-19 [39]. Open questions are still the evolving biology of the SARS-CoV-2 virus, and the development of new types of vaccines for a long-term immunity without disease and virus reproduction. Advances in this area will impact current IPC measures.

Our IPC strategy has been focusing on droplet/airborne precautions, requiring the universal use of N95 respirators in all hospital areas (by visitors, patients and staff), although the evidence for the additional protective effect of N95 respirators compared to medical face masks is weak [40]. Using face masks to stop respiratory transmission is less debatable [41]. The IPC strategy at the VGH as well as throughout Austria has also been heavily dependent on SARS-CoV-2 testing because tests were widely available, already in the early stage due to the pooling of multiple samples. In contrast, many American hospitals reported shortages of testing supplies and long turnaround times of test results at the beginning of the pandemic [34]. Testing allowed to reduce the risk of transmission to allow full hospital operation despite a lack of adequate infrastructure, e.g. not enough single rooms. However, testing should not delay healthcare. Whenever a timely test result could not be obtained (e.g. patients requiring an acute intervention), COVID-19 precautions were applied. For admission to a multi-occupancy room, a negative antigen test result sufficed (but concurrently, samples were taken for PCR analysis).

The combination of widespread PCR testing and the universal use of N95 respirators mitigated the increased transmission risk due to multi-occupancy rooms, an – at times – overburdened contact tracing team and compliance issues surrounding the universal mask mandate. This was reflected in only few nosocomial COVID-19 clusters despite nearly normal hospital operations. However, there has recently been an increase in the number of these clusters at the VGH, which coincided with a lifting of many previously applied preventive measures in the whole country. This shows that public health measures in the community directly impact case numbers at the hospital level, and the need to maintain IPC measures in healthcare institutions. Additionally, vigilance at the national level may be necessary to lower the risk for healthcare-associated transmission.

The preventive strategy of the VGH is an example of applying the framework for IPC in outbreak response, recently published by WHO [14]. This document gives very good instruction on how to provide safe care in hospitals during outbreaks, e.g. how to set up necessary infrastructure, how to educate and train HCWs and how to handle infectious patients. Studies comparing nosocomial COVID-19 clusters and patients’ outcomes in relation to different COVID-19 strategies in hospitals and countries may lead to additional insights to further improve the pandemic response.

This pandemic has exposed gaps in pandemic preparedness. We should see these failures as opportunities for future improvements, adapting the healthcare system in a way to address future emerging infectious diseases more effectively. Going forward, we need more international cooperation and coordination between governments, and a more temporal alignment of preventive public health measures across different countries, facilitated by the availability of high-quality data on infections [23, 42]. There is a need for contingency plans to prepare for PPE supply or staff shortages. Future hospitals have to be planned and constructed to better support infection prevention. Single rooms with sanitary units must be the standard as clearly supported by evidence for decreased transmission of multidrug-resistant microorganisms (MDRO) [43, 44]. Outpatient clinics will have to be built in a way to enable the separation of (potentially) infectious patients [45]. Additionally, technically appropriate ventilation systems are key for preventing the transmission or infectious aerosol particles [46]. This preparedness will ensure safe healthcare because infections caused by MDRO or emerging infections due to climate and environmental change plus associated migration will still be on the agenda even when COVID-19 is overcome. However, infrastructural and logistical solutions alone will not suffice. IPC strategies have to be backed by the population to ensure participation and compliance with the restrictions imposed on each individual. To achieve this, there is a need to improve communication strategies, adapting them to different socio-cultural contexts and combating systematic disinformation [47, 48]. The spread of “alternative facts” lead to skepticism and fear [49, 50]. There is a need to counter misinformation and fear by strengthening peoples’ confidence in science and improving health literacy [50, 51]. Knowledge and empowerment will lead to improved risk assessment and self-protection. To achieve this, basic hygiene concepts need more attention in school curricula, starting in primary schools, and IPC training should play a more prominent role in medical schools [52]. At the hospital level, regular IPC trainings (e.g. donning/doffing of PPE, hand hygiene) are key to ensure the correct use of the available resources. To maintain staff morale, there is a need for more support of healthcare workers so they can better cope with workload increases in a pandemic setting [38].

## Conclusions

The COVID-19 pandemic has sparked an enormous interest in infection prevention practices. This momentum should be harnessed to drive forward increased recognition for the field of hygiene and infection prevention, which also means allocating more funds to this area and investing in the training of additional IPC experts. Improvements in future hospital design and measures to increase health literacy will also be key. On a global level, governments need to cooperate more and pool efforts, which also means levelling up inequitable access to drugs, supplies and knowledge.

## Electronic supplementary material

Below is the link to the electronic supplementary material.

Supplementary Material 1

## Data Availability

The data used during the current study are available from the corresponding author on reasonable request.
