# Supplementary material for: COVID-19 patient and personal safety – lessons learnt for pandemic preparedness and the way to the next normal
Source: Antimicrob Resist Infect Control. 2023 Apr 1;12:27. doi: 10.1186/s13756-023-01231-1 (PMC10066952; doi:10.1186/s13756-023-01231-1)
Supplement: Supplementary file 1 — Supplementary Material 1 [file 13756_2023_1231_MOESM1_ESM.docx]

# Supplement


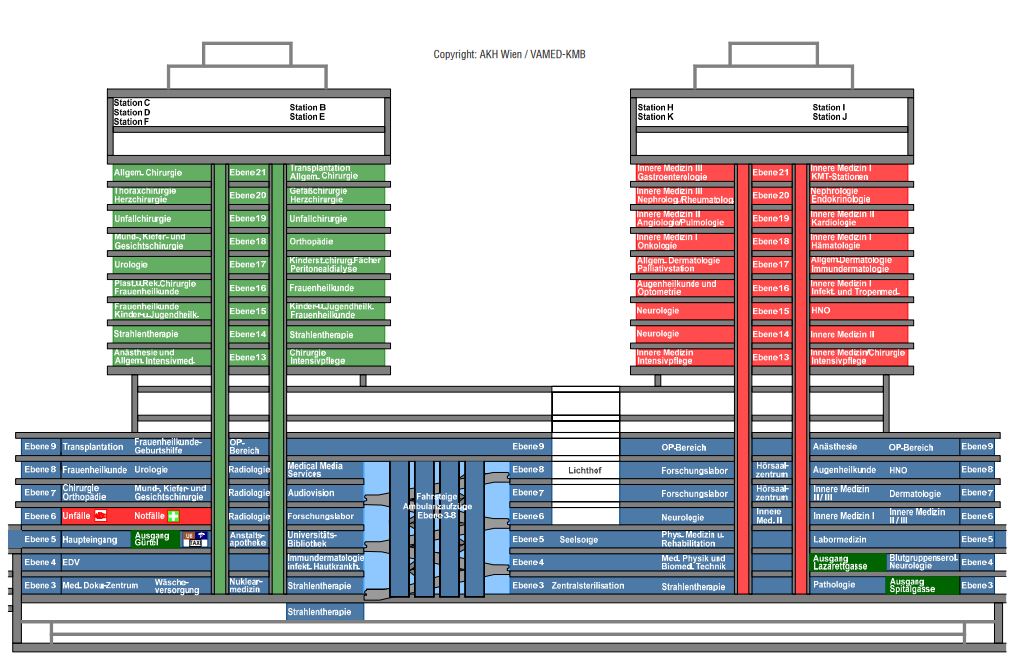


Figure S1. Layout plan of the Vienna General Hospital.

| **Table S1. Summary of infection prevention and control measures employed at the Vienna General Hospital and their evolution over time** | | | |
| --- | --- | --- | --- |
| **COVID-19 strategy** | | | |
| 01/2020 | Vienna General Hospital was operated as a non-COVID-19 facility; transferal of all COVID-19 cases to designated hospitals | | |
| 03-04/2020  Realization that it would not be possible to maintain VGH COVID-19 free in the long run | Integration of COVID-19 case management into routine care; strict separation between COVID-19 and non-COVID-19 wards | | |
| 11/2020  High case numbers in the community, resulting in more SARS-CoV-2 positive patients being admitted | Establishment of mixed wards | | |
| 05/2022  Low-incidence period, not requiring a whole intensive care unit dedicated to the care of COVID-19 patients | Establishment of mixed ICU wards | | |
| **Visitor regulations** | | | |
| 04/2020 | - General visitor ban with some exceptions (e.g. terminally ill patients) - Symptom screening and assessment of recent contact/travel history prior to gaining access | | |
| 10/2020 | - One visitor per patient per day: no COVID-19 symptoms - General visitor ban restricted to COVID-19 areas (with the same exceptions as above) | | |
| 03/2021 | No COVID-19 symptoms and   - Proof of previous infection (within the last 6 months) or - Negative SARS-CoV-2 test (antigen ≤ 48 hours, PCR ≤ 72 hours) | | |
| Austria introduced new rules as the basis for access restrictions | “3G” rule: proof of past infection or vaccination or negative antigen/PCR test  “2,5G” rule: past infection or vaccination or negative PCR test  “2G” rule: past infection or vaccination  “2G+”: past infection or vaccination PLUS negative PCR | | |
| 05/2021 | “3G” rule - access only with:   - Proof of previous infection or - Proof of previous vaccination or - Negative SARS-CoV-2 test (antigen ≤ 48 hours, PCR ≤ 72 hours) | | |
| 08/2021 | “3G” rule still in place, but shortened period of test validity: antigen ≤ 24 hours, PCR ≤ 48 hours | | |
| 09/2021 | “2,5G” rule - access only with:   - Proof of previous infection or - Proof of previous vaccination or - Negative test result (PCR ≤ 48 hours) – antigen tests no longer accepted | | |
| 11/2021 | “2G plus” rule - access only with:   - Proof of previous infection or - Proof of previous vaccination   PLUS   - Negative test result (PCR ≤ 48 hours) – antigen no longer accepted   Adolescents aged 12-17:   - Negative test result (PCR ≤ 48 hours); N95 respirator   Children aged 6-12:   - Negative test result (PCR ≤ 48 hours or antigen ≤ 72 hours); medical face mask   Children aged <6:   - Exempt from testing | | |
| 04/2022 | One visitor per patient per day  Access with a negative PCR test result (≤ 48 hours) | | |
| 08/2022 | Three visitors per patient per day  Access with a negative PCR test result (≤ 48 hours)  Exception: For critically ill patients and palliative care settings no test required | | |
| **Testing strategy for patients admitted to the VGH** | | | |
|  | **Patients presenting with acute conditions** | | **Elective admission** |
| 04/2020 | Immediately tested (PCR) and isolated until confirmed SARS-CoV-2 negative | | Universal pre-admission screening at designated testing site:   - Testing ≤ 48 hours before admission (PCR) - Home quarantine between testing and admission   Re-testing during hospitalization (PCR):   - 48 hours after admission - anytime in case of symptom onset - prior to AGPs - prior to transferal to other healthcare facilities |
| 07/2020 | No change | | Changes: the accepted time interval of the admission test was extended to 72 hours (PCR); re-testing during hospitalization was extended to 3 days after admission |
| 11/2020 | Additions:   - Testing prior to acute AGPs and surgery (antigen-test + PCR) - Symptomatic patients received an antigen test (if positive: PCR confirmation not necessary; if negative: additional PCR test & COVID-19 precautions until confirmed SARS-CoV-2 negative by PCR) - Asymptomatic patients received both antigen test + PCR test (negative antigen test allowed for transfer to multi-bed occupancy room; however, the patient should be equipped with an N95 respirator until confirmed negative by PCR) | | Changes:   - Re-testing 48-72 hours after admission (preferably using PCR, if antigen test conducted and result positive & patient asymptomatic: additional PCR) - Prior to elective AGPs (PCR) |
| 01/2021 | No change | | - Accepted time interval of admission test reduced to 48 hours again - Re-testing 3 days after admission (preferably using PCR; antigen tests also accepted) and then on a weekly basis - Exception for post-COVID-19 patients: re-tested only in case of symptom onset and after contact with a close case |
| 03/2021 | No change | | - Regular bi-weekly screening (preferably using PCR tests; antigen tests also accepted) - Exception: re-testing of vaccinated patients (at least 2 vaccine doses received) or post-COVID-19 patients (infection within the last 6 months) only once a week |
| 07/2021 | No change | | - Expansion of testing scheme to children > 6 years |
| 11/2021 | No change | | - Re-testing of patients without prior vaccination (preferably using PCR tests; antigen tests also accepted): twice (vaccinated patients) or 3 times (unvaccinated patients) per week |
| 03/2022 | No change | | - Changes for post-COVID-19 patients (infection within the last 8 weeks): no PCR test required for admission and screening with antigen-test (≤ 24 hours) instead; during hospitalization: no re-testing with PCR for 3 weeks following the infection; if needed antigen test should be conducted |
| 04/2022 | No change | | - Negative PCR result prior to elective admission controlled by treating physicians (not at entrance level) anymore |
| **Procedure after patient had contact with a confirmed case** | | | |
| 03/2020 | | Duration of isolation for 14 days following exposure   - Twice-daily temperature checks & symptom assessment (respiratory symptoms, etc.) - PCR testing after the contact became known, in case of symptom onset and prior to transferal to another hospital/long-term care facility | |
| 04/2020 | | Same as above; additional PCR test on day 4 after the exposure | |
| 07/2020 | | Duration of isolation reduced to 10 days  Modification of testing scheme: testing after the contact became known, on day 5 after exposure, in case of symptom onset or transferal | |
| 01/2021 | | Same as above; additional PCR test on day 10 after exposure if contact with a new virus variant was suspected | |
| 03/2021 | | Duration of isolation extended to 14 days, testing after the contact became known, on day 5 and 10 after exposure, and in case of symptom onset or transferal  Early termination possible on day 10 if   - Negative SARS-CoV-2 PCR/antigen test | |
| 04/2022 | | Duration of isolation reduced to 10 days, testing after the contact became known, on day 5 and 10  Early termination possible on day 5 if   - Negative SARS-CoV-2 PCR test 🡪 in this case no further test required on day 10   Isolation of all contacts, irrespective of prior vaccination (despite different national regulations) | |
| 08/2022 | | Same as above (despite the suspension of contact tracing in Austria) | |
| **Duration of isolation and transmission-based precautions for COVID-19 cases** | | | |
| 02/2020 | | Transferal to designated hospital; if not possible: isolation room with anteroom | |
| 03/2020 | | Isolation for 14 days after symptom onset/first positive test result  Termination possible if   - No fever for ≥ 48 hours, - No COVID-19 symptoms for ≥ 24 hours and - 2 neg. SARS-CoV-2 PCR tests from nasopharyngeal swabs taken 24 hours apart | |
| 04/2020 | | Isolation of COVID-19 cases in a single room, preferably with an anteroom and own toilet/shower | |
| 07/2020 | | Duration of isolation for at least 10 days after symptom onset/positive test  Termination possible if   - No symptoms for ≥ 48 hours and - 1 neg. SARS-CoV-2 PCR test or PCR with Ct-value >30 | |
| 03/2021 | | Duration of measures for 14 days after symptom onset/positive test  Mild course of disease: early termination of measures possible after 10 days if   - No symptoms for ≥ 48 hours and - 1 negative SARS-CoV-2 PCR/antigen test or PCR with Ct-value >30 | |
| 09/2021 | | Mild course of disease: early termination possible after 10 days if   - No symptoms for ≥ 48 hours and - 1 negative SARS-CoV-2 PCR test or Ct-value >30 | |
| 08/2022 | | Same as above (despite the end of compulsory home quarantine for SARS-CoV-2 positive individuals in Austria) | |
| **Screening of healthcare workers** | | | |
| 04/2020 | | Weekly testing (nasopharyngeal swabs, PCR “pool-testing”) | |
| 11/2020 | | Testing once (non-COVID-19 areas) or twice (areas with SARS-CoV-2 positive patients) per week (nasopharyngeal swabs, antigen test) | |
| 05/2021 | | Self-testing once per week; twice per week for unvaccinated HCWs (PCR gargle test) | |
| 11/2021 | | Self-testing twice per week (PCR gargle test)  HCWs without proof of vaccination/recovery: negative PCR test required on the first working day of the week, re-testing every 48 hours | |
| 01/2022 | | Addendum: HCWs with a recent SARS-CoV-2 infection self-tested using antigen tests instead of PCR (for 4 weeks after infection) | |
| 02/2022 | | Self-testing on a daily basis (PCR gargle test); at the same time suspension of contact tracing for HCWs  HCWs with a recent SARS-CoV-2 infection were exempt from self-testing for 3 weeks following the infection (unless symptoms re-occurred) | |
| 03/2022 | | New rules regarding early termination of home isolation:   - The day of the positive PCR-test counted as day 0; early termination of isolation possible on day 5 if   - no COVID-19 symptoms for at least 48 hours (e.g. fever) and   - Ct-value ≥ 30 or negative PCR - After 10 days no negative PCR result was required for the termination of isolation if   - no COVID-19 symptoms for at least 48 hours | |
| 04/2022 | | Interval of self-testing (PCR gargle test) reduced to twice per week | |
| 06/2022 | | Interval of self-testing (PCR gargle test) increased to three times per week | |
| 08/2022 | | Interval of self-testing (PCR gargle test) reduced to twice per week | |

| **Table S2. Summary of current IPC recommendations by ESCMID, WHO, ECDC and CDC** | |
| --- | --- |
| **Screening patients for SARS-CoV-2 in healthcare settings** | **ESCMID guideline(1):**  1. Universal pre-admission screening in areas with a high community transmission rate of SARS-CoV-2 and/or with low vaccination coverage (in settings where immunocompromised individuals are hospitalized, regardless of the transmission rate)  2. Re-testing (every 3 to7 days) during hospitalization only with a high level of community transmission or low vaccination rate, especially when other IPC measures are not appropriately in place (in settings where immunocompromised individuals are hospitalized, regardless of the transmission rate)  **ECDC(2):**  1. Universal pre-admission screening recommended in areas with documented community transmission  2. Re-testing 3-5 days after admission |
| **PPE for COVID-19 patient care** | **WHO(3):** medical mask, for AGPs: N95 or N99 respirator - they can also be used if they are widely available and costs are not an issue  **ECDC(4):** N95 respirator (acceptable alternative face mask if N95 respirator not available), for AGPs: N99 respirator  **CDC(5):** N95 respirator or higher (acceptable alternative face mask if N95 respirator not available) |
| **Duration of isolation of COVID-19 cases** | **ECDC(6):**  1. Mild/moderate COVID-19 (fully vaccinated):   - Resolution of fever, if present, for at least 24 hours and improvement of symptoms other than fever   &   - two negative SARS-CoV-2 rapid antigen detection test (RADT) or RT-PCR tests from respiratory specimens with a minimum 24-hour interval   OR   - six days after the onset of symptoms AND one negative RADT or RT-PCR test from respiratory specimen on day 6 or later   Advice to wear a face mask after ending isolation until day 10 after onset of symptoms  2. Severe COVID-19:   - Resolution of fever for at least 24 hours and improvement of symptoms other than fever   &   - two negative SARS-CoV-2 RADT or RT-PCR tests from respiratory specimens with a minimum 24-hour interval   OR   - minimum 14 and up to 20 days after the onset of symptoms   3. Immunocompromised:   - Resolution of fever, if present, for at least 24 hours and improvement of symptoms other than fever   &   - two consecutive negative SARS-CoV-2 RADT or RT-PCR tests from respiratory specimens with a minimum 24-hour interval   OR  20 days after the onset of symptoms, and ending isolation in conjunction with serial testing and consultation with an infectious disease specialist to determine the appropriate duration of isolation and precautions  **CDC(7):**  1. Mild COVID-19:   - Resolution of fever for at least 24 hours and improvement of symptoms other than fever   &   - at least five days after the onset of symptoms   Necessity to wear a face mask after ending isolation until day 10 after onset of symptoms (day 0 is the first day of symptoms)  2. Severe COVID-19:   - Duration of isolation for at least 10 up to 20 days after the onset of symptoms   3. Immunocompromised:   - Duration of isolation for at least 20 days |
| **Handling of contacts** | **WHO(8):**  1. Contacts who in the last 90 days have (i) completed the primary series vaccination, or (ii) have received a vaccine booster dose, or (iii) have reported a previous COVID-19 infection:   - No quarantine   2. Unvaccinated or without previous infection in the last 90 days:   - Home quarantine for 10 days - Early termination possible after 5 days if testing negative and no symptoms present   **ECDC(9):**  1. Fully vaccinated high-risk exposure:  For a period of 14 days after the last exposure:   - Get tested right away and self-quarantine at home until informed of a negative test result - If the test result is negative (particularly if an RADT was used) consider a second test two to four days afterwards - Daily symptom-check and physical distancing, self-isolation if symptoms occur   2. Unvaccinated (or partially vaccinated) high-risk exposure:   - Home quarantine until receipt of a negative RT-PCR test taken on day 10 or self-quarantine at home for 14 days.   **CDC(10):**  1. Fully vaccinated high-risk exposure:   - No quarantine - Get tested at least 5 days after the last exposure - Daily symptom-check and wearing of a well-fitting mask until 10 days after the last exposure   2. Unvaccinated (or partially vaccinated) high-risk exposure:   - Home quarantine for at least 5 days - Get tested at least 5 days after the last exposure - Daily symptom-check and wearing of a well-fitting mask until 10 days after the last exposure |

## References

1. Carrara E, Ong DSY, Hussein K, Keske S, Johansson AF, Presterl E, et al. ESCMID guidelines on testing for SARS-CoV-2 in asymptomatic individuals to prevent transmission in the health care setting. Clinical microbiology and infection : the official publication of the European Society of Clinical Microbiology and Infectious Diseases. 2022;28(5):672-80.

2. European Centre for Disease Prevention and Control. COVID-19 testing strategies and objectives. Stockholm: ECDC; 2020.

3. World Health Organization. Mask use in the context of COVID-19 2020 [20.07.2022]. Available from: <https://www.who.int/publications/i/item/advice-on-the-use-of-masks-in-the-community-during-home-care-and-in-healthcare-settings-in-the-context-of-the-novel-coronavirus-(2019-ncov)-outbreak>.

4. European Centre for Disease Prevention and Control. Personal protective equipment (PPE) needs in healthcare settings for the care of patients with suspected or confirmed 2019-nCoV, 07.02.2020 Stockholm: ECDC; 2020 [10.08.2022]. Available from: <https://www.ecdc.europa.eu/en/publications-data/personal-protective-equipment-ppe-needs-healthcare-settings-care-patients>.

5. Centers for Disease Control and Prevention. Interim Infection Prevention and Control Recommendations for Healthcare Personnel During the Coronavirus Disease 2019 (COVID-19) Pandemic 2022 [19.07.2022]. Available from: <https://www.cdc.gov/coronavirus/2019-ncov/hcp/infection-control-recommendations.html#anchor_1604360721943>.

6. European Centre for Disease Prevention and Control. Guidance on ending isolation of people with COVID-19, third update. Stockholm; 2022.

7. Centers for Disease Control and Prevention. Ending Isolation and Precautions for People with COVID-19: Interim Guidance 2022 [18.07.2022]. Available from: <https://www.cdc.gov/coronavirus/2019-ncov/hcp/duration-isolation.html>.

8. World Health Organization. Contact tracing and quarantine in the context of COVID-19: interim guidance, 6 July 2022 2022 [19.07.2022]. Available from: <https://www.who.int/publications/i/item/WHO-2019-nCoV-Contact_tracing_and_quarantine-2022.1>.

9. European Centre for Disease Prevention and Control. Contact tracing in the European Union: public health management of persons, including healthcare workers, who have had contact with COVID-19 cases – fourth update 2021 [15.07.2022]. Available from: <https://www.ecdc.europa.eu/en/covid-19-contact-tracing-public-health-management>.

10. Centers for Disease Control and Prevention. Quarantine and Isolation 2022 [19.07.2022]. Available from: <https://www.cdc.gov/coronavirus/2019-ncov/your-health/quarantine-isolation.html>.
